# Application of Rapid Biological Indicators Coupled With Auto-Reader for the Quality Assurance of Surgical Instruments After Sterilization at a Cardiac Hospital in Bangladesh

**DOI:** 10.7759/cureus.19428

**Published:** 2021-11-10

**Authors:** Sifat U Zaman, Israt Sadia, Nawzia Yasmin, Kamrun Nahar Islam, M Mushfequr Rahman, Ahsanul Haq, Taslin Jahan Mou, Nafisa Azmuda, Mainul Haque, Nihad Adnan

**Affiliations:** 1 Department of Microbiology, Jahangirnagar University, Dhaka, BGD; 2 Division of Infection Prevention and Control, Medlife Healthcare Limited, Dhaka, BGD; 3 Department of Infection Control, Labaid Cardiac Hospital, Dhaka, BGD; 4 Department of Public Health, State University of Bangladesh, Dhaka, BGD; 5 Department of Microbiology, University of Chittagong, Dhaka, BGD; 6 Department of Microbiology, BRB Hospitals Limited, Dhaka, BGD; 7 Department of Statistics, Gonoshasthaya-RNA Molecular Diagnostic & Research Center, Dhaka, BGD; 8 Department of Pharmacology and Therapeutics, National Defence University of Malaysia, Kuala Lumpur, MYS

**Keywords:** ethylene oxide, steam sterilizer, infection prevention control, surgical site infections, hospital-acquired infections

## Abstract

Background

Sterilization failure is one of the main reasons for surgical site infections (SSIs). The biological indicator (BI) test is the most reliable test to check sterilization efficiencies. But 48 hours BI test result makes the monitoring process time-consuming. Rapid BI testing can be time demanding in this regard. Therefore, the objective is to determine the importance of rapid BI monitoring for the quality assurance of sterile surgical instruments.

Methods

This study was conducted in the Labaid Cardiac Hospital, Bangladesh from April 1, 2021, to July 8, 2021. A total of 100 steam and 100 ethylene oxide (EO) rapid BIs and an auto reader incubator were used to conduct this research. Quick BI of steam and EO were used once per day and tested by the auto reader. Later, all the tested BIs were incubated for 48 hours by a conventional incubator to confirm the auto reader's rapid BI test results.

Result

All the EO BI results were found negative, but the BI was found positive twice in steam sterilization. Surgical items of those two loads were re-sterilized. Again, after checking the BI result, the items were released. All BIs except positive steam rapid BIs were found with no growth after 48 hours of incubation for cross-checking of auto reader results. In positive rapid BI of steam, growth was found after 48 hours of incubation.

Conclusion

When sterilization failure occurred, process recall could not be possible at that time if rapid BI tests were not performed. So, integration of a rapid BI test with an auto reader can save the patient from critical SSI.

## Introduction

Infection prevention and control (IPC) is considered to be the primary disease containment method in healthcare settings [1]. Sterilization of surgical instruments is one of the prime requisites of surgical site infection (SSI) prevention [2, 3]. Some surgical instruments or devices are designed and recommended to be reused, known as "multiple use medical devices," after adequate decontamination by proper cleaning and sterilization procedure [4].

Steam sterilization versus ethylene oxide sterilization

Many sterilization methods are available and frequently used worldwide. Steam sterilization, also known as the moist-heat sterilization method, is a frequently used method of sterilization, uses high temperature saturated steam of 121°C-135°C. This method is regarded as the most common, cost-effective, and robust method among other sterilization methods [2, 3]. On the other hand, ethylene oxide (EO) is used in many regions for heat-labile products and low-temperature sterilization. Based on the wide range of applications in sterilization and development of compatible medical devices, the significance of using EO has emerged recently [5]. Improper sterilization of surgical instruments or medical devices carries a high risk of infection [6]. In low and middle-income countries, inadequate or inappropriate sterilization of surgical instruments is one of the common factors resulting in healthcare-associated infections than in developed countries [6, 7]. Therefore, quality control and assurance are essential in the sterilization procedure to ensure sterility [8].

Biological indicator as a test for sterilization monitoring

The biological indicator (BI) test is considered one of the most reliable tests done for sterilization monitoring. There are spore strips that contain Bacillus spp. or Geobacillus spp. spore as the BIs. As spores of these bacterial species can withstand high temperatures than the vegetative ones, the sterilizer's efficiency is measured by destroying the spore in a BI after completion of sterilization [8, 9]. Daily use of the BI test is recommended by the international regulatory institution [10].

Two types of BI test methods have generally been used, i.e., conventional culture-based method and rapid BI test. The former takes 24-48 hrs to obtain the sterilization result, whereas the latter method takes 20 minutes to 4 hrs [11]. When a rapid BI test is conjugated with an auto-reader, the sterilization process can be recalled within hours if the sterilization process fails [12]. On the other hand, if conventional culture-based methods are used, sterilization recall takes time, thus increasing the chances of using improperly sterilized equipment in surgery.

To the best of researchers' knowledge, no such study was conducted in Bangladesh. In this study, 200 rapid BIs were used in a cardiac hospital in Bangladesh to monitor the quality assurance of the sterilization of surgical instruments. Failure in a few sterilization processes was found using rapid indicators, which indicated the importance of integrating rapid BIs in the sterilization units.

## Materials and methods

Study design

A cross-sectional, descriptive, experimental, and exploratory study was designed to conduct this research. The study was conducted in Central Sterile Supply Department (CSSD) Laboratory, Infection Prevention and Control Department, Labaid Cardiac Hospital, Dhaka, Bangladesh, and performed from April 1, 2021, to July 8, 2021.

Study population

**Figure 1:**
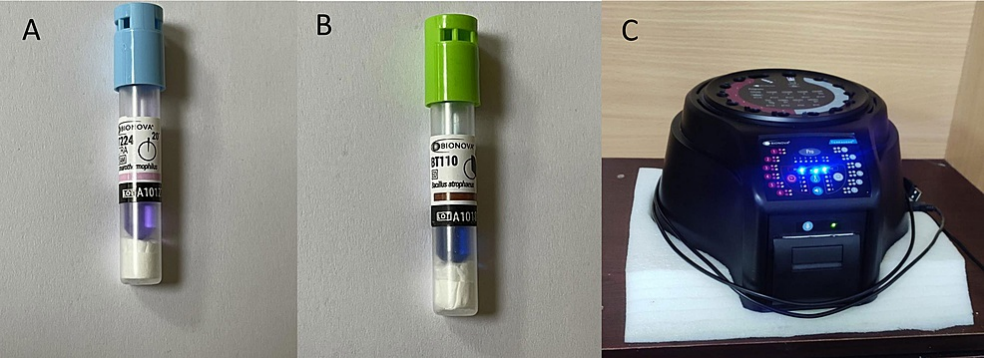
Instruments used in the study, (A) Rapid Steam Biological Indicator (BI224), (B) Rapid EO Biological Indicator (BI110), and (C) Bionova Auto-Reader Incubator IC10/20FR. EO: Ethylene oxide.

One hundred self-contained rapid BIs for steam Bionova BT224 (Terragene, Argentina) (*Geobacillus stearothermophilus* ATCC 7953 spore) (Figure 1A) and one hundred self-contained rapid BIs for EO Bionova BT110 (Terragene, Argentina) (*Bacillus atrophaeus* ATCC 9372 spore) (Figure 1B), in total 200 processed BIs were used to conduct this research (Figure 1). Bionova Auto-Reader Incubator IC10/20FR (Terragene, Argentina) (Figure 1C) was used for the rapid biological indicator result, where fluorescent technology was used for rapid detection (Figure 1). The rapid BIs and IC10/20FR auto reader is certified by US FDA.

Sampling method

In Bangladesh, very few hospitals and clinics are using both steam and EO sterilizers. Among the hospital setting, Labaid Cardiac Hospital agreed to use its facilities for sample collection. Thus purposive sampling technique was employed to collect the samples from Labaid Cardiac Hospital for this research to be carried out. Each BI of steam and EO was used 1-2 pcs per day and tested by Bionova Auto Reader, which gives steam BI results within 20 minutes, and EO BI results within four hours.

Laboratory testing and data collection

After completing steam sterilization and EO sterilization of medical devices, the tested BIs of each sterilization process were brought out from the sterilizer and put into the auto reader machine for rapid results, and results were recorded. Later, the steam BIs were incubated for 48 hours at 56°C and EO BI at 37°C in conventional incubators, according to the manufacturer's instructions [13].

Statistical analysis

The data was entered in spreadsheets, and later, the data were imported into IBM SPSS Statistics 22 software (SPSS Inc., Chicago, IL, USA) for analysis. Thereby, a descriptive exploratory statistical analysis was conducted to determine the BI experiment result.

## Results

**Figure 2:**
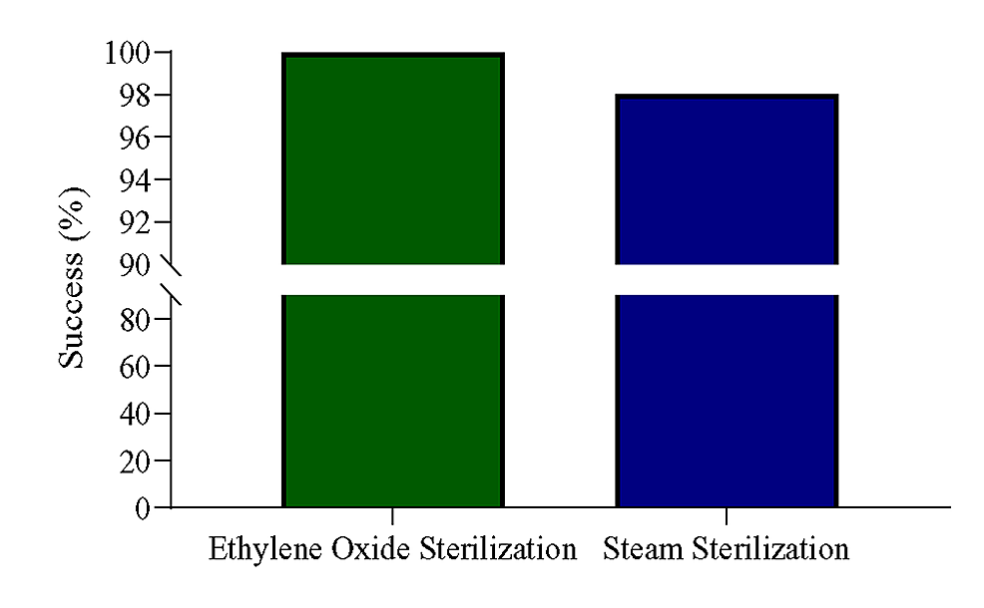
Success and failure percentage of EO sterilization and steam sterilization detected by the auto-reader. EO: Ethylene oxide.

The study results were obtained through the auto-reader and the sterilized success was 100% in the EO process, whereas, 98% with steam-sterilization at the end of the experiment (Figure 2), The auto-reader observed two steam sterilization process failures, one on May 10, 2021, and another on June 26, 2021. These two batches were recalled immediately and sterilized again. The equipment of these batches was only used after reconfirmation of sterilization by the auto-reader.

**Figure 3:**
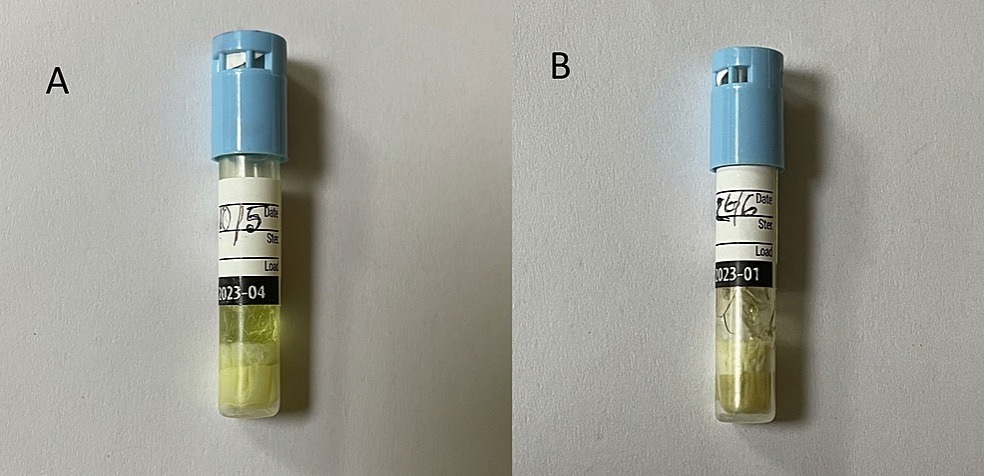
Steam sterilization failure indicated by biological indicators incubated at 56°C for 48 hours, (A) May 10, 2021, and (B) June 26, 2021.

All the rapid BI test tubes were incubated at appropriate conditions, and it was found that all 198 tubes, except for two failed batches, showed no growth (blue color). Growth in the failed batches (Figure 3) was confirmed by the color change of the media from blue to yellow (Figures 3A-3B).

Thus, when the accuracy of the auto-reader was compared with the conventional culture-based method, it was revealed that the results of the auto-reader were 100% precisely confirmed within a short time. In contrast, culture-based methods required a longer time to indicate the effectiveness of the sterilization processes (Table 1).

**Table 1:** Accuracy of the rapid biological indicator test result. EO: Ethylene oxide.

Biological Indicator	Rapid Readout Result by the Auto-Reader	Conventional Method by 48 Hours Incubation
EO Biological Indicator	100 (Negative)	100 (No Growth)
Steam Biological Indicator	98 (Negative)	98 (No Growth)
	2 Positive	2 (Growth)

## Discussion

Multiple research studies conducted by Dancer SJ et al. (2012), Lu WP et al. (2012), and Tosh PK et al. (2011) reported that incidents of infections were amplified because of inadequate and improper sterilization process of surgical instruments. Consequently, it was evident from all three studies the extent of suffering the patients would have had to endure [14-16]. In our study, all the EO sterilization cycles from April 1 to July 8, 2021, were successful, indicating that the reusable medical devices were properly sterilized by the EO gas' sterilizing agent. All the EO BI results were found to be negative by the Bionova Auto Reader (Figure 2). Moreover, no growth was observed after 48 hours of incubation at 37°C (Table 1).

Sterilization failure occurred twice during the study period, but it was negative in the rest 98 cases during the steam sterilization process. The auto-reader initially detected the sterilization failure (Figure 2) which was later confirmed by the culture-based method (Figure 3A-3B and Table 1). Although, globally steam sterilization technique is known as the most effective sterilization technique for reusable surgical instruments, there are several reports of steam sterilization failure. The study conducted by Panta G et al. (2019) revealed a high proportion of steam sterilization failure incidents at several hospitals in Nepal [17]. There are similar reports from studies done by Miranzadeh MB et al. (2011) and Okemwa KA et al. (2014) [18,19]. In a study by Sickder HK et al. (2017), the overall SSI prevalence rate in a tertiary hospital was 14.13%, and the most common isolated pathogens were *Staphylococcus aureus* (41.9%), *Escherichia coli* (30.8%), and *Enterococcus* spp. (12%) [20].

Regular surveillance systems must be implemented to provide reliable data to determine SSIs prevalence, incidence, and distribution [21]. Observations from this study indicate possible lacunae in performing steam sterilization and help in ascertaining efficient solutions. Rapid BI test combined with auto-reader provides an opportunity to re-sterilize the surgical instruments if the sterilization process fails, within a short span of time. Thus, increases the possibility of saving patients from the high risk of SSIs. Thereby, reduces morbidity, mortality, hospital stay, and healthcare cost. Although this study has found EO sterilization more efficient than steam sterilization, proper precaution should be maintained during handling and cleaning the EO sterilizers.

Study limitations

Due to time and fund constraints, a small sample size was taken to conduct this research. Moreover, only one hospital was included as a study site. The inclusion of more hospitals in the rural and urban areas could show the actual sterilization practice and determine the efficacies of the rapid BIs when combined with an auto-reader.

## Conclusions

This study will alert the healthcare staff regarding possible deficiencies in practicing steam sterilization and provide a better solution to avoid unexpected outcomes. The use of rapid BI tests combined with auto reader machines is a rapid and reliable solution to confirm the sterilization processes, thus can significantly reduce SSIs.

## References

[1] Ocampo W, Geransar R, Clayden N (2017). Environmental scan of infection prevention and control practices for containment of hospital-acquired infectious disease outbreaks in acute care hospital settings across Canada. Am J Infect Control.

[2] Stryja J, Sandy-Hodgetts K, Collier M (2020). Surgical site infection: Prevention and management across health-care sectors. J Wound Care.

[3] Al-Benna S (2012). Infection control in operating theatres. J Perioper Pract.

[4] (2021). Decontamination and Reprocessing of Medical Devices for Health-care Facilities. http://apps.who.int/iris/bitstream/handle/10665/250232/9789241549851-eng.pdf.

[5] Mendes GC, Brandão TR, Silva CL (2007). Ethylene oxide sterilization of medical devices: a review. Am J Infect Control.

[6] Weber DJ, Rutala WA (2013). Assessing the risk of disease transmission to patients when there is a failure to follow recommended disinfection and sterilization guidelines. Am J Infect Control.

[7] (2016). WHO. Report on the burden of endemic healthcare-associated infection worldwide. http://apps.who.int/iris/bitstream/10665/80135/1/9789241501507_eng.pdf..

[8] Palenik CJ, Burke FJ, Coulter WA, Cheung SW (1999). Improving and monitoring autoclave performance in dental practice. Br Dent J.

[9] Pflug IJ, Odlaug TE (1986). Biological indicators in the pharmaceutical and medical device industry. J Parenter Sci Technol.

[10] Ling ML, Ching P, Widitaputra A, Stewart A, Sirijindadirat N, Thu LT (2018). APSIC guidelines for disinfection and sterilization of instruments in health care facilities. Antimicrob Resist Infect Control.

[11] Vesley D, Langholz AC, Rohlfing SR, Foltz WE (1992). Fluorimetric detection of a Bacillus stearothermophilus spore-bound enzyme, α-d-glucosidase, for the rapid indication of flash sterilization failure. Appl Environ Microbiol.

[12] da Fonseca EP, Pereira-Junior EA, Palmier AC, Abreu MH (2021). A description of infection control structure in primary dental health care, Brazil. Biomed Res Int.

[13] Rutala WA, Jones SM, Weber DJ (1996). Comparison of a rapid readout biological indicator for steam sterilization with four conventional biological indicators and five chemical indicators. Infect Control Hosp Epidemiol.

[14] Dancer SJ, Stewart M, Coulombe C, Gregori A, Virdi M (2012). Surgical site infections linked to contaminated surgical instruments. J Hosp Infect.

[15] Lu WP, Lin GX, Shi S, Dong JH (2012). Simultaneously high prevalences of hepatitis B and C virus infections in a population in Putian County, China. J Clin Microbiol.

[16] Tosh PK, Disbot M, Duffy JM (2011). Outbreak of Pseudomonas aeruginosa surgical site infections after arthroscopic procedures: Texas, 2009. Infect Control Hosp Epidemiol.

[17] Panta G, Richardson AK, Shaw IC, Chambers S, Coope PA (2019). Effectiveness of steam sterilization of reusable medical devices in primary and secondary care public hospitals in Nepal and factors associated with ineffective sterilization: a nation-wide cross-sectional study. PLoS One.

[18] Miranzadeh MB, Sabahibidgoli M, Afshar M, Zarjam R (2013). Study on the biological monitoring of steam sterilizer in Kashan governmental hospitals during 2011. J Appl Sci Environ Sanit.

[19] Okemwa KA, Kibosia CJ, Nyamagoba H (2014). Instrument sterilization practices and monitoring in private and public dental clinics in Eldoret, Nakuru and Kisumu municipalities in Western Kenya. J Kenya Den Assoc.

[20] Sickder HK, Lertwathanawilat W, Sethabouppha H, Viseskul N (2017). Prevalence of surgical site infection in a tertiary-level hospital in Bangladesh. Int J Nat Soc Sci.

[21] Fan Y, Wei Z, Wang W (2014). The incidence and distribution of surgical site infection in mainland China: a meta-analysis of 84 prospective observational studies. Sci Rep.

